# LncRNA-RMST Functions as a Transcriptional Co-regulator of SOX2 to Regulate miR-1251 in the Progression of Hirschsprung's Disease

**DOI:** 10.3389/fped.2022.749107

**Published:** 2022-03-07

**Authors:** Lingling Zhou, Zhengke Zhi, Pingfa Chen, Chunxia Du, Binyu Wang, Xiang Fang, Weibing Tang, Hongxing Li

**Affiliations:** ^1^General Surgery Department, Children's Hospital of Wujiang District, Suzhou, China; ^2^Department of Pediatric Surgery, Children's Hospital of Nanjing Medical University, Nanjing, China; ^3^Intensive Care Unit, The Fourth Affiliated Hospital of Nantong University, The First People's Hospital of Yancheng, Yancheng, China

**Keywords:** Hirschsprung's disease, lncRNA-RMST, miR-1251, SOX2, AHNAK

## Abstract

Hirschsprung's disease (HSCR) is a congenital disorder characterized by the absence of enteric neural crest cells (ENCCs). LncRNA rhabdomyosarcoma 2-associated transcript (RMST) is essential for the growth and development of neuron. This study aimed to reveal the role of RMST in the pathogenesis of HSCR. The expression level of RMST, miR-1251, SOX2, and AHNAK was evaluated with qRT-PCR or western blot. CCK-8 and transwell assays were applied to detect cell proliferation and migration. CHIP and RIP assays were applied to determine the combination relationship between SOX2 and promoter region of miR-1251 or RMST and SOX2, respectively. Dual-luciferase reporter assay was performed to confirm miR-1251 targeted AHNAK. As results have shown, RMST was downregulated in the aganglionic colon of HSCR patients. The knockdown of RMST attenuated cell proliferation and migration significantly. MiR-1251, the intronic miRNA of RMST, was also low expressed in HSCR, but RMST did not alter the expression of miR-1251 directly. Furthermore, SOX2 was found to regulate the expression of miR-1251 *via* binding to the promoter region of miR-1251, and RMST strengthened this function by interacting with SOX2. Moreover, AHNAK was the target gene of miR-1251, which was co-regulated by RMST and SOX2. In conclusion, our study demonstrated that RMST functioned as a transcriptional co-regulator of SOX2 to regulate miR-1251 and resulted in the upregulation of AHNAK, leading to the occurrence of HSCR. The novel RMST/SOX2/miR-1251/AHNAK axis provided potential targets for the diagnosis and treatment of HSCR during embryonic stage.

## Graphical Abstract

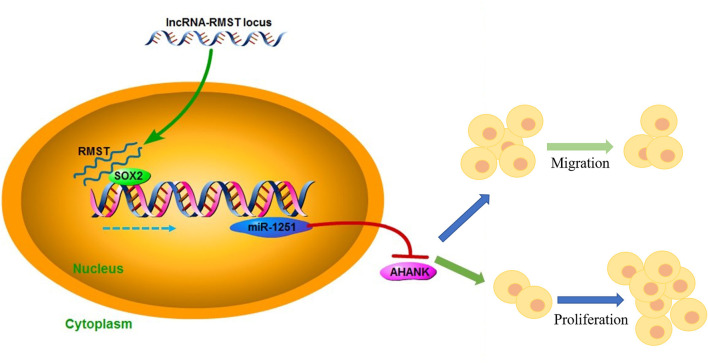

## Introduction

Hirschsprung's disease (HSCR), an enteric neuropathy, is characterized by the absence of gangliocytes in the distal colon (1, 2). It is caused by the impaired migration and proliferation of enteric neural crest cells (ENCCs) during the 5th to 12th weeks of embryogenesis (3). HSCR usually occurs in about 1/5,000 neonates, while the incidence rate of females is about a quarter of males (4). Current etiological studies show that HSCR is a complicated disorder involving multiple genetic factors, including RET, GDNF, GFRA1, EDNRB, and PHOX2B (5, 6). However, these genes could only explain a portion of the known cases, so further research is needed.

Long non-coding RNAs (lncRNAs) are increasingly considered to be important players in cellular biological processes, such as cell proliferation and migration, by affecting gene expression at nearly all levels (7, 8). LncRNA TPTEP1 was reported to inhibit the proliferation of non-small cell lung cancer cells through abating miR-328-5p expression (9). In renal cell carcinoma, lncRNA00312 attenuated cell proliferation and migration significantly (10). In addition, lncRNA DRAIC was found to regulate cell proliferation and migration in HSCR by affecting the miR-34a-5p/ITGA6 pathway (11). The silence of AFAP1-AS1 promoted HSCR progression by acting as an endogenous RNA to absorb miR-195 (12). Moreover, LOC100507600 is proved to participate in the development of HSCR through regulating BMI1 expression in a miR128-1-3p–dependent manner (13). However, the functions of lncRNAs in HSCR remain largely unknown.

RMST has been authenticated as a critical role during the neuronal differentiation (14, 15). Briefly, Ng et al. (16) induced differentiation of the ReN-VM neural stem cells using N2B27 medium, and 7 days later, the control group yielded TUJ1^+^ and MAP2^+^ neurons, while very few positive stained cells were observed in RMST knockdown group. In addition, RMST could promote the activation of microglial cells by activating TAK1-mediated NF-κB signaling (17). From the microarray analysis in our previous study, we found that RMST was downregulated in HSCR, indicating that RMST might play a key role in the progression of HSCR (18). Interestingly, miR-1251, which was transcribed from the same genomic region as RMST, was also downregulated in aganglionic segment. However, we found that RMST did not regulate miR-1251 directly. There might be other regulatory mechanisms to be uncovered.

Sex determining region Y (SRY)-box 2 (SOX2) is implicated in transcriptional regulation (19, 20). For example, SOX2 has been shown to regulate multiple malignant processes of breast cancer through the SOX2/miR-181a-5p, miR-30e-5p/TUSC3 axis (21). More importantly, SOX2 is closely related to the nervous system, and the terminal differentiation of postmitotic olfactory neurons was directly regulated by SOX2 (22). Numerous evidence has indicated that the downregulation of SOX2 attenuated cell growth and migration obviously (23, 24). Herein, SOX2 was found low expressed in HSCR and was predicted to bind to the promoter region of miR-1251 through bioinformatics analysis. Furthermore, RMST could enhance the regulation of SOX2 on downstream genes by interacting with SOX2 (25).

In this study, we demonstrated that RMST was downregulated in HSCR aganglionic colon and inhibited cell proliferation and migration by functioning as a transcriptional co-regulator of SOX2 to regulate miR-1251 in the progression of HSCR.

## Materials and Methods

### Clinical Information

This study was approved by the Institutional Ethics Committee of Children's Hospital of Nanjing Medical University (approval number: 201703057), and the experiments were carried out according to approved guidelines. In total, 32 aganglionic colon tissues were collected from patients who accepted radical operation of HSCR (age: 126.8 ± 14.31 days; gender: male 26, female 6) in Children's Hospital of Nanjing Medical University. In total, 32 controls matched with cases on age and gender were randomly picked out from isolated patients (age: 103.1 ± 10.47 days; gender: male 25, female 7) who underwent surgery for intussusception or incarcerated strangulated inguinal hernia (without enteric nervous malformation). The clinical features are also shown in Table 1. Tissues were harvested and stored at −80°C immediately after surgery. All HSCR patients were diagnosed through pathological analysis. Written informed consent from all participants was obtained.

**Table 1:** Clinical characteristics of study population.

**Variable**	**Control**	**HSCR**	***p***
Age(d, mean±SE)	103.1 ± 10.47	126.8 ± 14.31	0.19^a^
**Gender**
Male	25	26	0.76^b^
Female	7	6	

a *Student's test*.

b *Two-sided chi-squared test*.

### Microarray Analysis

The microarray analysis was performed as our previous study described (18). Briefly, the Agilent human lncRNA Array v3.0 (4 × 180k format) was designed for profiling human lncRNAs. GeneSpring v12.0 software (Agilent, USA) was employed for the raw data summarization, normalization, and quality control. For the selection of dysregulated lncRNAs, the threshold value of ≥2- and <2-fold change and a Benjamini–Hochberg corrected *p*-value of 0.05 were adopted. Kangchen Bio-tech (Shanghai, China) conducted this microarray analysis.

### Quantitative Real-Time PCR

To isolate total RNA from tissues and cells, Trizol reagent (Invitrogen Life Technologies Co, USA) was applied. qRT-PCR was employed to detect RMST, miR-1251, and AHNAK expression level by using SYBR (Takara Bio, Japan) reactions on Light Cycler 480 (Roche, Switzerland) according to the manufacturer's protocol. GAPDH and U6 were applied as an internal control for mRNA and miRNA detection, respectively. The expression quantity was analyzed with the 2^−ΔΔCT^ method. Primer sequences are shown in Table 2.

**Table 2:** Primer sequences for quantitative RT-PCR.

**Target gene**	**Primer sequence (5^**′**^-3^**′**^)**
GAPDH	F: GCACCGTCAAGGCTGAGAAC
	R: GGATCTCGCTCCTGGAAGATG
U6	F: CTCGCTTCGGCAGCACA
	R: AACGCTTCACGAATTTGCG
RMST	F: ACTTCTGAGTGGTATGCTGCT
	R: GGATGGTGGTTTTGATGTTTC
SOX2	F: TTGCTGCCTCTTTAAGACTAGGA
	R: CTGGGGCTCAAACTTCTCTC
AHNAK	F: TACCCTTCCTAAGGCTGACATT
	R: TTGGACCCTTGAGTTTTGCAT
miR-1251	F: ACACTCCAGCTGGG ACTCTAGCTGCCAAA
	R: CTCAACTGGTGTCGTGGAGTCGGCAATTCAGTTGAG AGCGCCTT
promoter region of miR-1251	F: TGGACAAGCTGAAGATATGGACA
	R: TGACCTCGATGGCAGTGATG

### Western Blotting

RIPA lysis buffer (Beyotime, Shanghai, China) was applied to extract total proteins from colon tissues and cultured cells. BCA Protein Assay Kit (Beyotime, Shanghai, China) was used to detect protein concentration. The same amount of total proteins were isolated in 10% SDS-PAGE, subsequently transferred to PVDF membranes, and then blocked with fat-free milk for 1 h. At 4°C, primary antibodies were used for incubation overnight. Afterwards, corresponding secondary antibodies were added for 2 h of incubation at room temperature. Finally, the membranes were imaged *via* ECL and Western blot detection reagents (Thermo Fisher Scientific, MA, USA). Primary antibodies including anti-AHNAK (1:1,000, SC134252), anti-SOX2 (1:1,000, SC17320X), and anti-GAPDH (1:1,000, SC47724) were obtained from Santa Cruz (CA, USA). The corresponding secondary antibodies were obtained from Beyotime (Shanghai, China).

### Chromatin Immunoprecipitation

By using ChIP Assay Kit (Thermo Fisher Scientific, Shanghai, China), ChIP was implemented in accordance with the operating instructions. First, cross-linked chromatin was sonicated into around 200- to 1,000-bp fragments. Anti-SOX2 was used to immunoprecipitate the chromatin. Goat immunoglobulin G (IgG, ab172730) was employed to be the negative control. PCR was performed using SYBR Green Mix (Takara Bio, Japan). The primer sequences are shown in Table 2.

### Cell Culture and Transfection

SH-SY5Y and 293T cell lines were acquired from ATCC. Cells were cultured at 37°C, 5% CO_2_ condition using DMEM (Hyclone, USA) culture medium containing 10% FBS, 100 U/ml penicillin, and 100 μg/ml streptomycin. The inhibitor of miR-1251 (a chemically modified RNA single strand), siRNAs of RMST, SOX2, and AHNAK, and the corresponding negative controls were synthesized by Genechem (Shanghai, China). Transfection experiments were conducted by using Lipofectamine 2000 Reagent (Invitrogen, USA).

### Cell Proliferation Assay

To test the cell viability, cell counting kit-8 (CCK-8; Dojindo, Japan) was employed. After transfection, cells were cultured in 96-well-plates for 24–48 h and subsequently incubated with CCK-8 reagent for 1–2 h. Eventually, the OD value at 450 nm was detected by the TECAN infinite M200 Multimode microplate reader (Tecan, Mechelen, Belgium). Each assay was conducted independently in triplicate.

### Cell Migration Assay

Transwell chambers were placed above a 24-well-plate. After transfection around 24–48 h, cells were resuspended with serum-free medium to 1 × 10^6^ cells/ml. About 1 × 10^5^ cells were seeded to the upper chamber. Five hundred microliters of complete culture medium containing FBS was added to the lower chamber. Then 24–48 h later, 4% paraformaldehyde was applied to fix the lower chamber cells and then crystal violet staining solution was used to stain cells. Cells that migrated to the lower chamber were counted and imaged using an inverted microscope (×20, five fields were randomly selected for counting). All experiments were conducted in triplicate.

### Dual-Luciferase Reporter Assay

pGL3-AHNAK-WT and pGL3-AHNAK-MUT were constructed by inserting the predicted 3′-UTR sequence of AHNAK binding to miR-1251 and the mutated sequence into the pGL3 promoter vector (Genechem, Shanghai, China). For reporter assay, cells were plated into 24-well-plates and transfected with 100 ng of pGL3-AHNAK-WT and pGL3-AHNAK-MUT, 50 nM miR-1251 mimics, and negative control using Lipofectamine 2000. Renilla luciferase vector pRL-SV40 (5 ng) was transfected into cells as control. Based on the obtained ratio, the activation degree of target reporter genes in different sample was compared.

### Statistical Analysis

GraphPad Prism 7.0 (GraphPad Software, USA) was adopted for data analysis. Between two groups, *t*-test was applied to determine the statistically significant differences, while the comparison among multiple groups was performed *via* one-way ANOVA. All data were presented as the mean ± SEM. *P* < 0.05 was considered to be statistically significant.

## Results

### RMST and miR-1251 Were Downregulated in HSCR Patients

**Figure 1:**
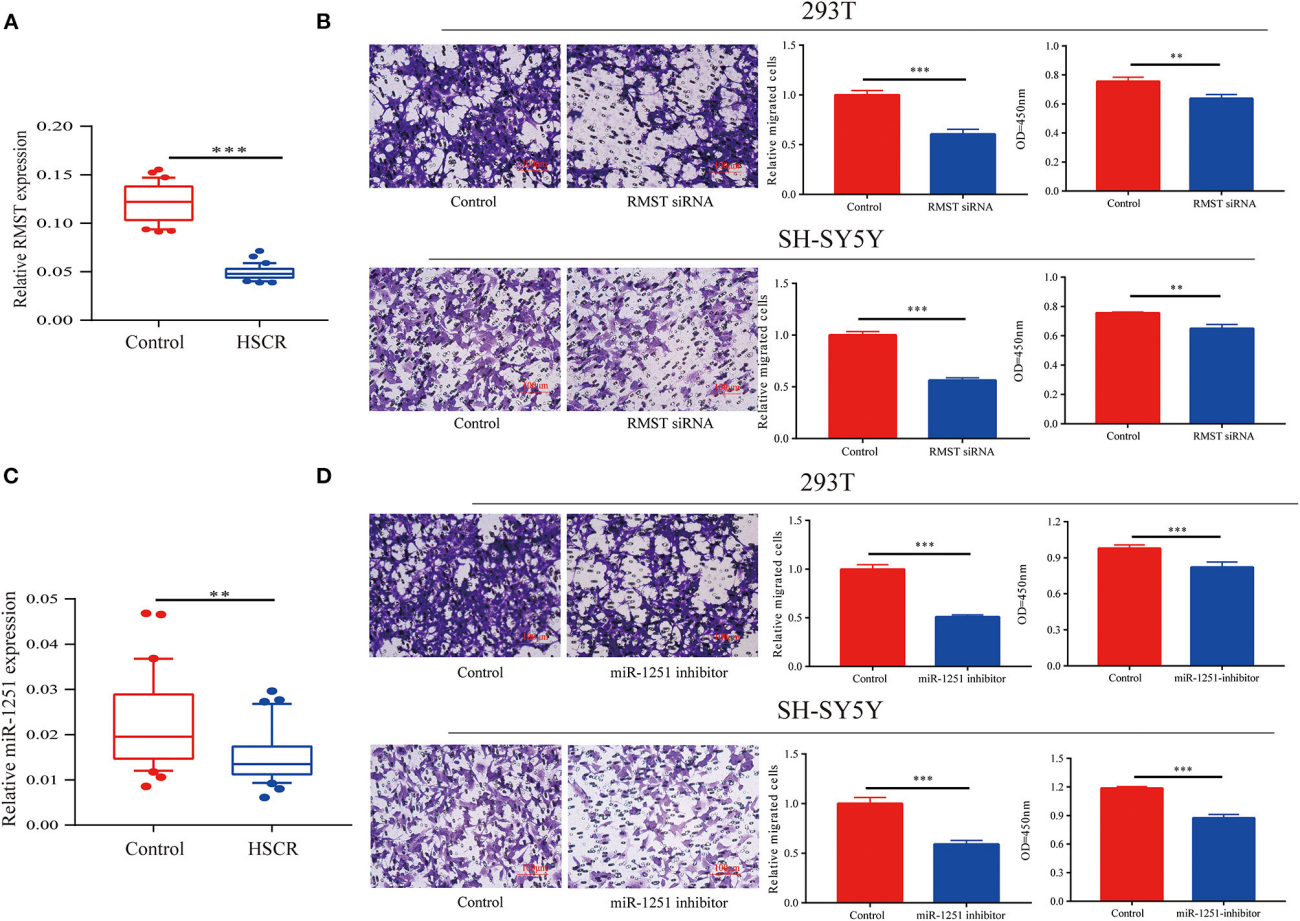
RMST and miR-1251 were downregulated in HSCR patients. **(A)** RMST was downregulated obviously in aganglionic tracts of HSCR patients than control ones. **(B)** Transwell and CCK-8 assays showed that cell migration and proliferation was inhibited when RMST was knocked down in 293T and SH-SY5Y cells. **(C)** MiR-1251 was low expressed obviously in aganglionic colon tissues of HSCR patients compared with controls. **(D)** Cell proliferation and migration was attenuated obviously when miR-1251 was knocked down in 293T and SH-SY5Y cells. **p < 0.01, ***p < 0.001.

By qRT-CR, we found RMST was obviously downregulated in aganglionic colon segments compared with normal controls (Figure 1A). Receiver operating characteristic (ROC) curve analysis showed that RMST could serve as a molecular marker for the prognosis of HSCR (Supplementary Figure 1G). Transwell and CCK-8 assays showed that the knockdown of RMST inhibited both SH-SY5Y and 293T cells' proliferation and migration (Figure 1B). We also found the RMST intronic transcript miR-1251 was downregulated in aganglionic tracts (Figure 1C). When cells were transfected with miR-1251 inhibitor, the cell migration and proliferation was also attenuated significantly (Figure 1D).

### miR-1251 Was Transcriptionally Regulated by SOX2

**Figure 2:**
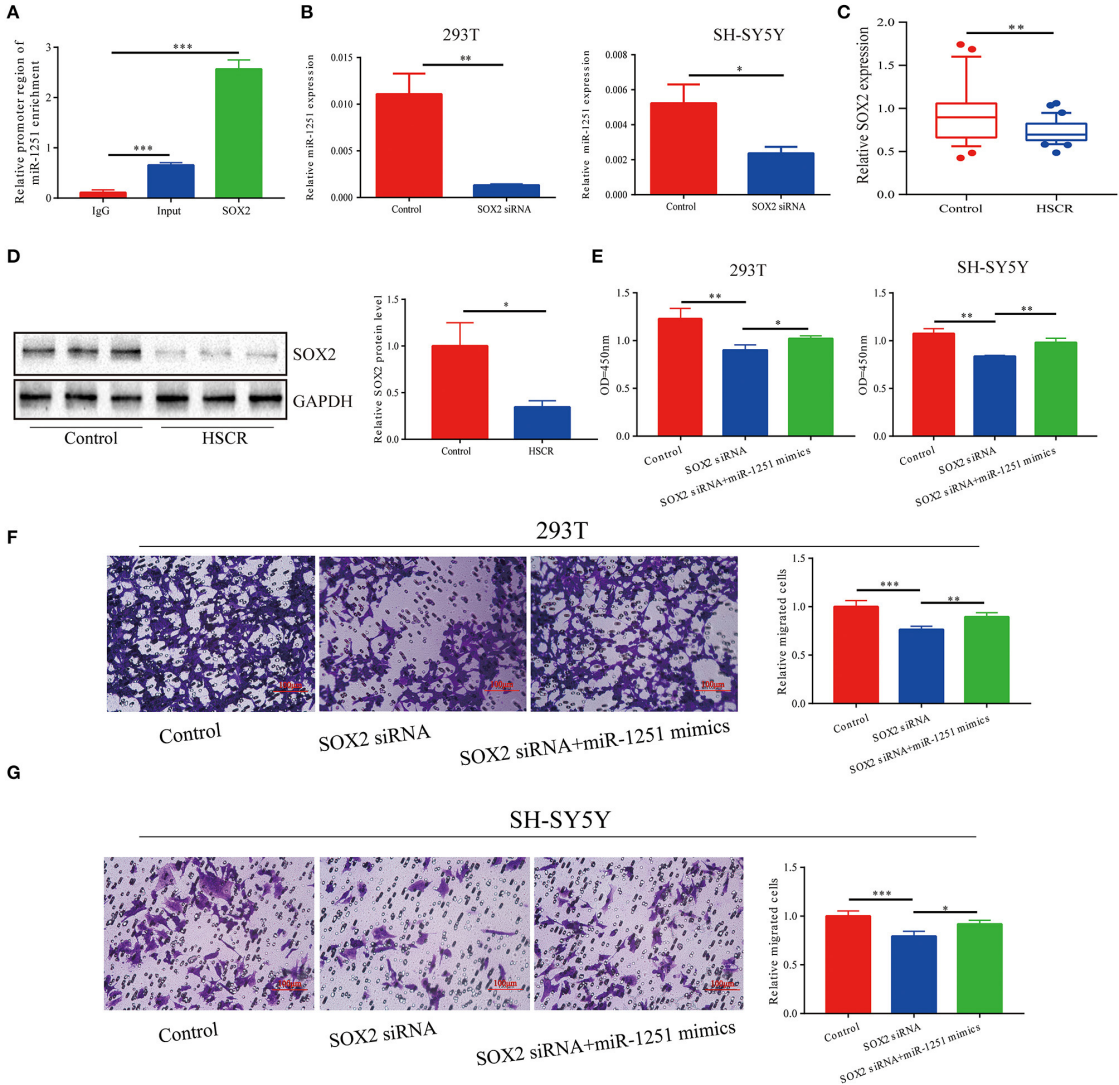
miR-1251 was transcriptionally regulated by SOX2. **(A)** CHIP assay showed that SOX2 could bind to the promoter region of miR-1251. **(B)** After downregulating SOX2, the expression of miR-1251 was reduced. **(C,D)** SOX2 was downregulated in HSCR patients at both mRNA and protein level. **(E–G)** When 293T and SH-SY5Y cells were transfected with si-SOX2, cell migration, and proliferation was inhibited, but the upregulation of miR-1251 could partially reverse it. *p < 0.05, **p < 0.01, and ***p < 0.001.

Since miR-1251 is transcribed from the same genomic locus as RMST, we suspected that RMST might regulate the expression level of miR-1251. However, there was no significant change on miR-1251 expression level after the downregulation of RMST, indicating RMST did not regulate miR-1251 directly (Supplementary Figure 1B). Moreover, SOX2 was predicted to bind with the 2-kbp upstream promoter region of miR-1251 using Promoter Scan (http://www.ncbi.nlm.nih.gov/Class/NAWBIS/Modules/DNA/dna21b.html) (Supplementary Figure 1F). The binding relationship was confirmed by the ChIP experiment (Figure 2A), and the silence of SOX2 decreased miR-1251 expression significantly (Figure 2B). In aganglionic colon segments, SOX2 was low expressed at both mRNA and protein levels compared with normal controls (Figures 2C,D). When cells were transfected with SOX2 siRNA, cell proliferation and migration was weakened obviously, while upregulating miR-1251 could reverse it partially (Figures 2E–G).

### RMST Functioned as a Co-regulator of SOX2

**Figure 3:**
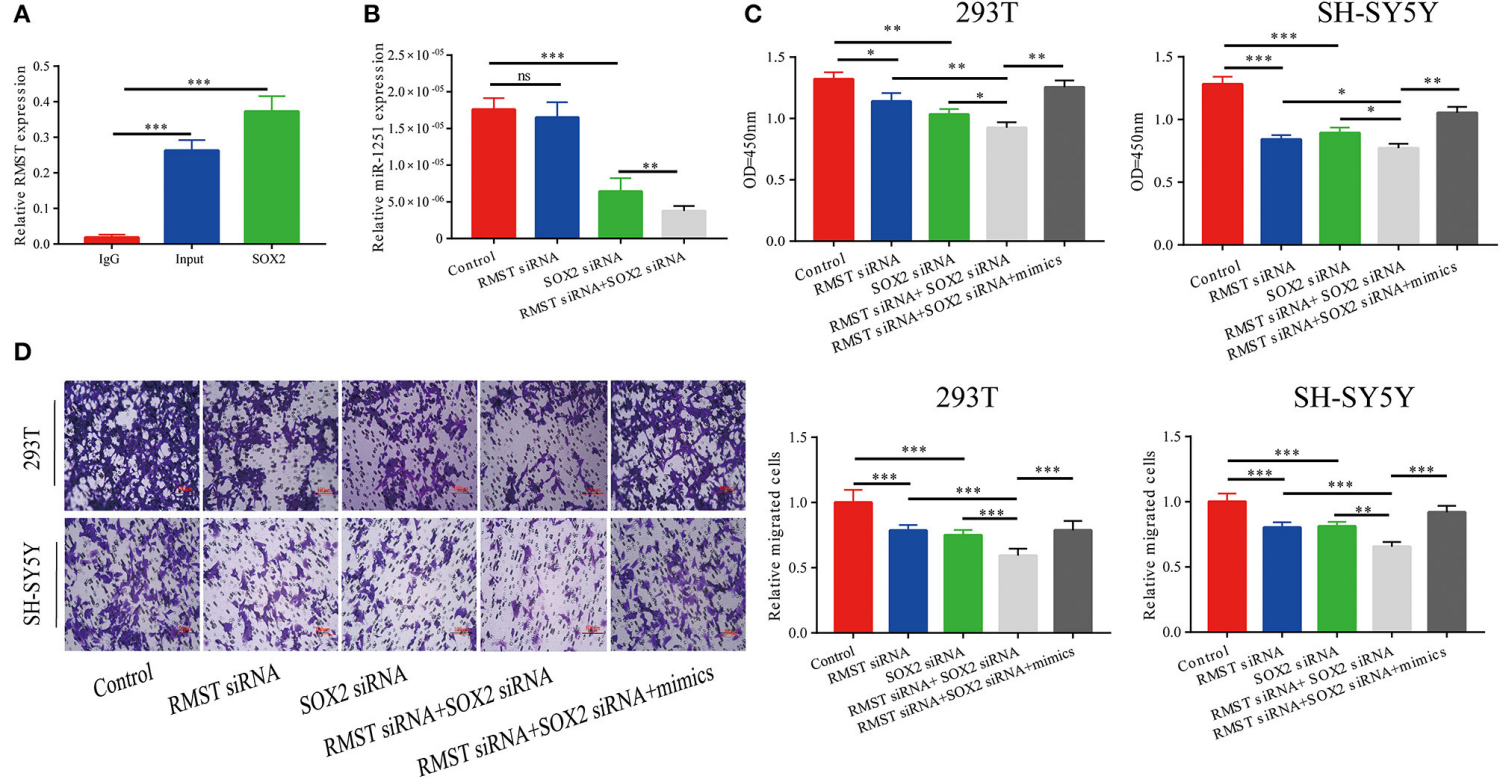
RMST functioned as a co-regulator of SOX2. **(A)** RIP assay confirmed RMST could bind to SOX2 protein. **(B)** The expression of miR-1251 was downregulated in “SOX2 siRNA” group and was reduced more in “RMST siRNA+SOX2 siRNA” group, but was not changed significantly in “RMST siRNA” group compared with the control group. **(C,D)** CCK-8 and transwell assays revealed that when the expression of RMST and SOX2 were both knocked down, the cell proliferation and migration was more weakened than just downregulated RMST or SOX2 alone. However, the upregulation of miR-1251 could partly reverse the inhibitory effects of “RMST siRNA+SOX2 siRNA” on cell proliferation and migration. *p < 0.05, **p < 0.01, and ***p < 0.001.

The RIP assay revealed that RMST interacted with SOX2 protein, indicating RMST might function through SOX2 (Figure 3A). As shown in Figure 3B, miR-1251 was downregulated after cells were transfected with SOX2 siRNA and the expression of miR-1251 was much lower in cells co-transfected with RMST siRNA and SOX2 siRNA. CCK-8 and transwell assays have shown that the knockdown of both RMST and SOX2 attenuated cell proliferation and migration more obviously compared with just downregulating RMST or SOX2 alone. Meanwhile, the upregulation of miR-1251 partially reversed the combined suppressive effects of si-RMST and si-SOX2 (Figures 3C,D).

### AHNAK Was the Target Gene of miR-1251

**Figure 4:**
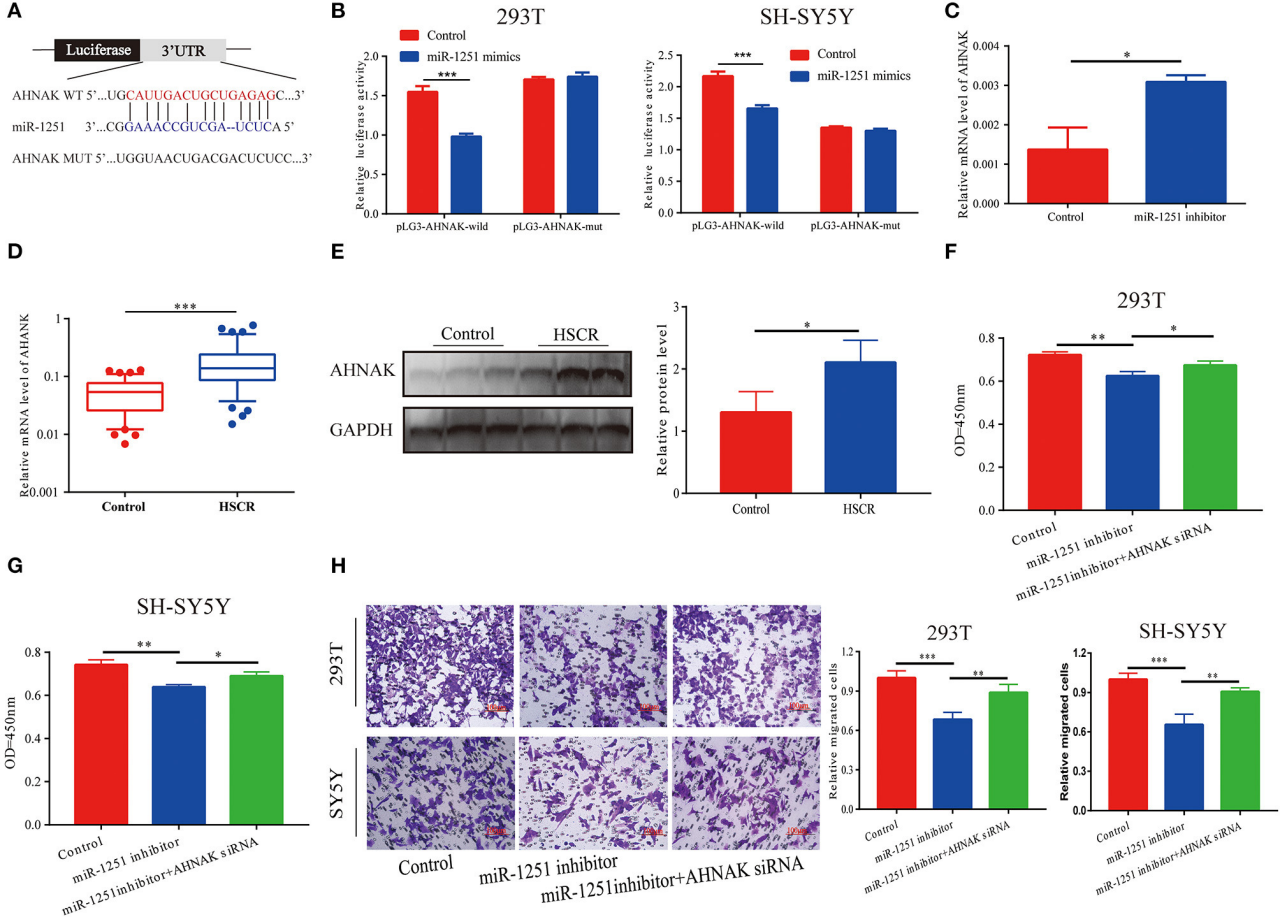
AHNAK was the target gene of miR-1251. **(A)** The schematic diagram of binding sites between miR-1251 and AHNAK. **(B)** The luciferase activity was abated obviously when transfected with miR-1251 mimics and pLG3-AHNAK-wild compared with control; however, the luciferase activity was not changed significantly when transfected with miR-1251 mimics and pLG3-AHNAK-mut in 293T and SY5Y cells. **(C)** When miR-1251 was knocked down, the mRNA level of AHNAK was raised. **(D,E)** AHNAK was upregulated in stenotic tracts of HSCR patients at mRNA and protein level compared with control tracts. **(F–H)** The downregulation of miR-1251 inhibited cell migration and proliferation, but the knockdown of AHNAK could partially reverse it in 293T and SH-SY5Y cells. *p < 0.05, **p < 0.01, and ***p < 0.001.

MiR-1251 was predicted to interact with the 3′-UTR region of AHNAK (Figure 4A). Compared with the control group, the luciferase activity was significantly decreased when cells were co-transfected with miR-1251 mimics and pGL3-AHNAK-WT plasmid (Figure 4B). In 293T cells, the knockdown of miR-1251 increased the expression of AHNAK (Figure 4C). In aganglionic tracts, AHNAK was overexpressed at both mRNA and protein levels (Figures 4D,E). As rescue experiment results have shown, the reduction of AHNAK could partially reverse the influence of miR-1251 inhibitor on cell migration and proliferation (Figures 4F–H).

### RMST Functioned as a Transcriptional Co-regulator of SOX2 to Inhibit miR-1251 and Raise AHNAK Expression

**Figure 5:**
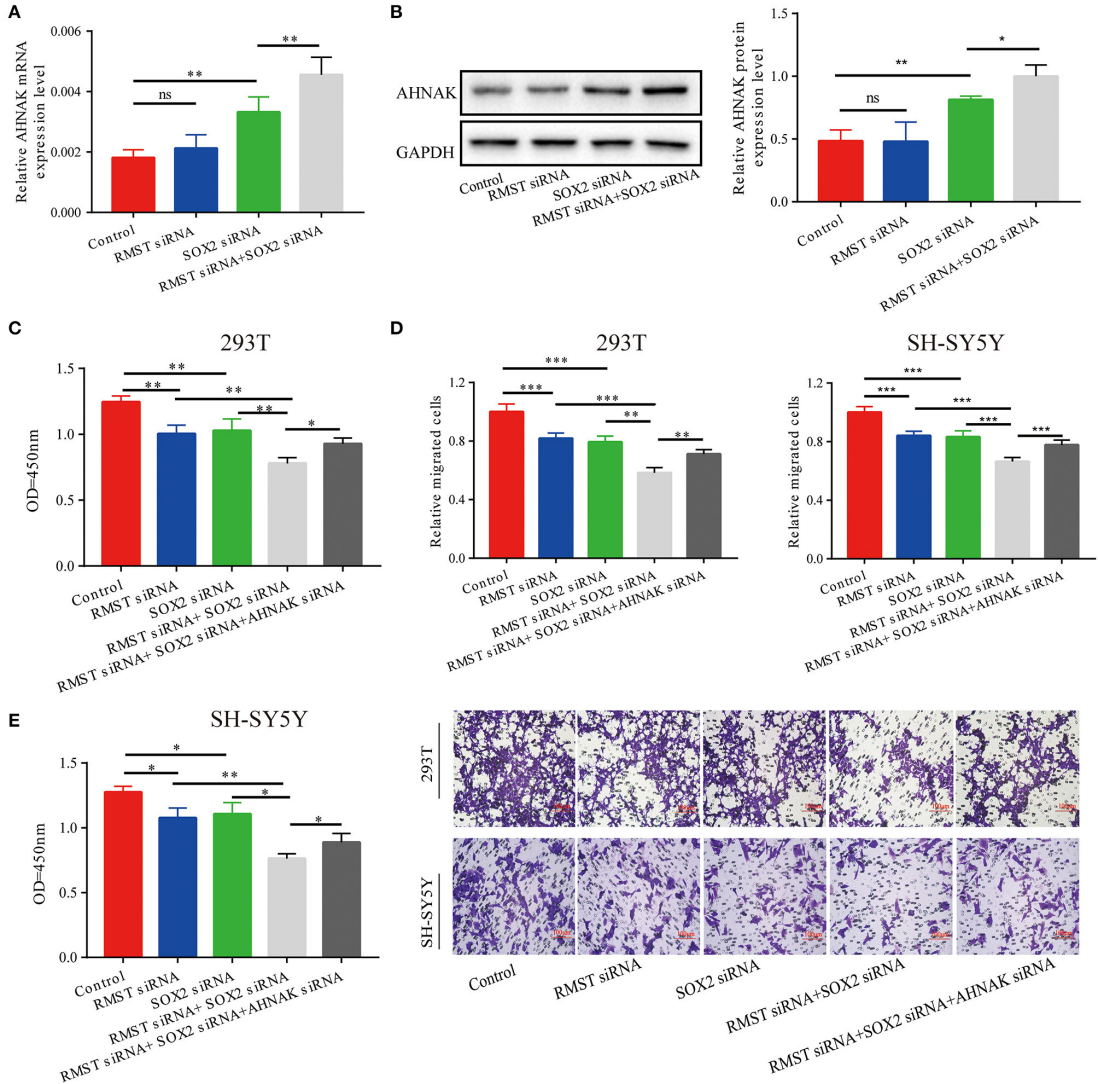
RMST functioned as a SOX2 transcriptional co-regulator to inhibit miR-1251 and raise AHNAK expression. **(A,B)** When cells were transfected with RMST siRNA, the mRNA, and protein expression of AHNAK was not changed obviously; however, the transfection of SOX2 siRNA raised the expression of AHNAK and the downregulation of both RMST and SOX2 increased this more. **(C,D)** The cell proliferation and migration was inhibited in “RMST siRNA” or “SOX2 siRNA” groups compared with control and was inhibited more obviously in “RMST siRNA+SOX2 siRNA” group. **(E)** However, the simultaneous downregulation of AHNAK could partially alleviate the inhibitory effects caused by the knockdown of both RMST and SOX2. ns, *p* ≥ 0.05, *p < 0.05, **p < 0.01, and ***p < 0.001.

Combined with the aforementioned results, we hypothesized that RMST enhanced the regulation of SOX2 on miR-1251 and then promoted the expression of AHNAK. The expression of AHNAK showed no significant difference between the RMST siRNA group and the control group. However, the expression of AHNAK was increased when SOX2 was knocked down (Figures 5A,B). Furthermore, the expression of AHNAK was much lower in “RMST siRNA+SOX2 siRNA” group than in “SOX2 siRNA” group, indicating that RMST upregulated the expression of AHNAK *via* acting as a SOX2 transcriptional co-regulatory factor (Figures 5A,B). Afterwards, the rescue experiments were set up, and we found the combined inhibitory effects of “RMST siRNA and SOX2 siRNA” on cell proliferation and migration could be partially alleviated by simultaneously downregulating the expression of AHNAK (Figures 5C,D).

## Discussion

Recently, more and more ncRNAs, especially miRNA and lncRNA, have been found to play critical roles in epigenetic regulation and take part in the occurrence and development of numerous diseases (26–28). Although there have been some reports about ncRNAs in HSCR, its mode of action and mechanism still require further studies (29–34). In this study, we detected that RMST and the intronic miR-1251 were downregulated in the aganglionic tracts of HSCR patients for the first time. The diagnostic value of RMST was also assessed *via* ROC. However, the number of samples was relatively not large enough and more population samples are needed for further validation. The knockdown of RMST inhibited cell proliferation and migration obviously, indicating that RMST might play a key role in the progression of HSCR.

MiR-1251 was first demonstrated as a potential prognostic marker in head and neck squamous cell carcinoma (35). The role of miR-1251 in other diseases is still unclear. Herein, we found that the cell proliferation and migration was significantly inhibited by knocking down miR-1251, suggesting that miR-1251 might also be involved in the pathogenesis of HSCR through inhibiting the proliferation and migration of ENCCs. Although miR-1251 was derived from the intronic region of RMST, we found that RMST did not regulate the expression of miR-1251 directly. There might be other regulatory mechanisms.

According to the bioinformatical analysis, SOX2, a transcription factor, was predicted to bind to the promoter region of miR-1251, and we performed ChIP assay to confirm this binding relationship. SOX family, such as SOX10, has been proved to be related to the pathogenesis of HSCR (36), but there are few reports about the role SOX2 exerts in HSCR. SOX2 has been reported to regulate the proliferation and differentiation of peripheral nerve cells in the peripheral nervous system (37). Moreover, SOX2 is closely related to the development of embryonic neural tube and neural crest cells (38–40). In the present study, we found that SOX2 was significantly downregulated in aganglionic tracts of HSCR patients. Furthermore, the downregulation of SOX2 significantly inhibited cell proliferation and migration, while the upregulation of miR-1251 could partially reverse it. These findings elucidated that SOX2 might be critical for the biological function of ENCCs by regulating miR-1251.

LncRNAs are known to bind with certain proteins to influence the function target proteins (41). Zhang et al. (42) found LINC00319 contributed to AML leukemogenesis *via* elevating SIRT6 through FUS-dependent pathway. LncRNA OCC-1 was verified to suppress cell growth through binding to and destabilizing HuR protein in colorectal cancer (43). RMST has been reported to bind to SOX2, and we also confirmed the relationship by using RIP assay in the present study, suggesting that RMST might function as a co-regulator of SOX2 in HSCR. We found that miR-1251 expression was reduced more when RMST and SOX2 were both knocked down compared with silencing SOX2 alone. Furthermore, the inhibition of cell proliferation and migration was more obviously when downregulating RMST and SOX2 than just decreasing RMST or SOX2. However, the enforced expression of miR-1251 partially reversed the combinative inhibitory effects, indicating that RMST functioned by acting as a transcriptional co-regulator of SOX2 to enhance the regulation of SOX2 on miR-1251.

MiRNAs generally exert their functions by degrading the target genes (44). We found that AHNAK, a kind of scaffold protein, was the target gene of miR-1251. The upregulation of AHNAK has been shown to impair cell proliferation and migration (45). In this study, we elucidated the upregulation of AHNAK in HSCR. Moreover, we found that AHNAK expression was not changed significantly when RMST was silenced. The knockdown of SOX2 increased the expression of AHNAK, and the downregulation of RMST strengthened this effect. Combined with the aforementioned, we revealed that RMST could regulate the expression of AHNAK and exerted its roles through RMST/SOX2/miR-1251/AHNKA axis.

Taken together, our study demonstrated that the downregulation of RMST inhibited cell proliferation and migration in HSCR. In terms of mechanism, RMST functioned as a transcriptional co-regulator of SOX2 to regulate the expression of AHNAK by strengthening the regulatory effect of SOX2 on miR-1251. The novel RMST/SOX2/miR-1251/AHNAK pathway might be helpful for the understanding of the pathogenesis of HSCR and the development of targeted therapy for HSCR.

However, this study still has some limitations. When the expression of RMST was decreased alone, the cell proliferation, and migration was also inhibited. Whether RMST has other regulatory patterns requires further investigation. In addition, *in vivo* experiments are also needed for further study.

## Data Availability Statement

The datasets presented in this study can be found in online repositories. The names of the repository/repositories and accession number(s) can be found in the article/Supplementary Material.

## Ethics Statement

This study was approved by the Institutional Ethics Committee of Children's Hospital of Nanjing Medical University (approval number: 201703057), and the experiments were conducted in accordance with the principles of the Declaration of Helsinki. All parents of patients had provided written informed consent in the study. Written informed consent to participate in this study was provided by the participants' legal guardian/next of kin.

## Author Contributions

HL and WT: designed the project. LZ, ZZ, PC, and BW: performed the experiments. LZ, CD, and XF: analyzed the data. ZZ and HL: wrote the paper. All authors discussed the results and commented on the article.

## Funding

This work was supported by the Natural Science Foundation of China (NSFC 81701493) and General project of Nanjing Health and Family Planning Commission (YKK19103).

## Conflict of Interest

The authors declare that the research was conducted in the absence of any commercial or financial relationships that could be construed as a potential conflict of interest.

## Publisher's Note

All claims expressed in this article are solely those of the authors and do not necessarily represent those of their affiliated organizations, or those of the publisher, the editors and the reviewers. Any product that may be evaluated in this article, or claim that may be made by its manufacturer, is not guaranteed or endorsed by the publisher.

## Supplementary Material

Supplementary Figure 1 **(A)** The transfection efficiency of si-RMST in 293T and SH-SY5Y cells. **(B)** There was no significant change in miR-1251 expression in 293T and SH-SY5Y cells when transfected with si-RMST. The transfection efficiency of miR-1251 inhibitor **(C)**, si-AHNAK **(D)**, and oe-AHNAK **(E)** in 293T and SY5Y cells. **(F)** SOX2 was predicted to bind with the 2-kbp upstream promoter region of miR-1251. **(G)** The ROC analysis of RMST. ns, *p* ≥ 0.05, ^**^*p* < 0.01, and ^***^*p* < 0.001.

Supplementary Figure 2 The flowchart of bioinformatics analysis steps. The binding relationship between RMST and SOX2 was demonstrated by reference and RIP assay. Promoter Scan was applied to predict the binding relationship between SOX2 and miR-1251 promoter region. MicroRNA database was employed to predict the target gene of miR-1251.
