# Supplementary figures and images for: LncRNA-RMST Functions as a Transcriptional Co-regulator of SOX2 to Regulate miR-1251 in the Progression of Hirschsprung's Disease
Source: Front Pediatr. 2022 Mar 7;10:749107. doi: 10.3389/fped.2022.749107 (PMC8936393; doi:10.3389/fped.2022.749107)

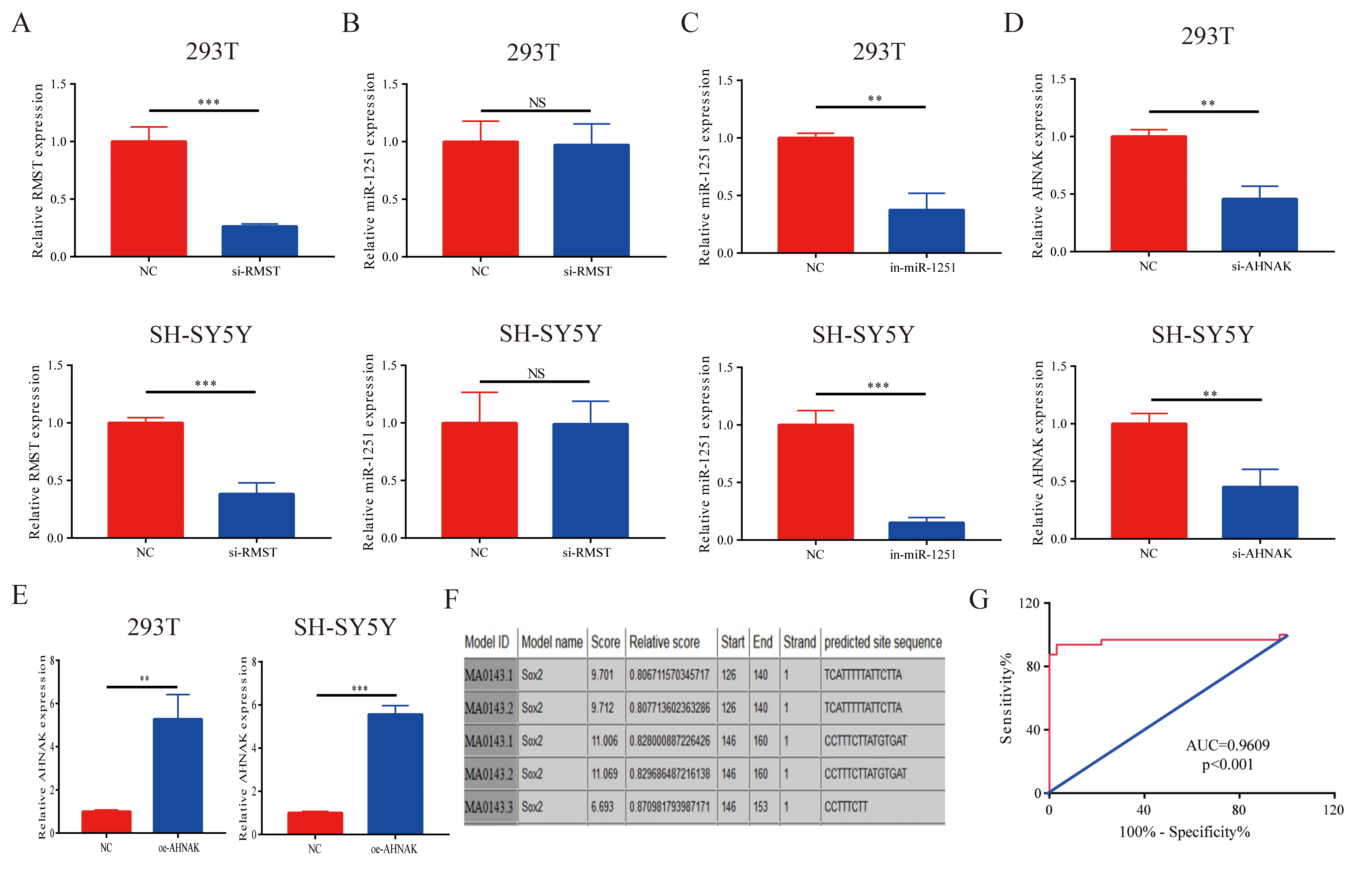

Supplement: Supplementary Figure 1 — (A) The transfection efficiency of si-RMST in 293T and SH-SY5Y cells. (B) There was no significant change in miR-1251 expression in 293T and SH-SY5Y cells when transfected with si-RMST. The transfection efficiency of miR-1251 inhibitor (C), si-AHNAK (D), and oe-AHNAK (E) in 293T and SY5Y cells. (F) SOX2 was predicted to bind with the 2-kbp upstream promoter region of miR-1251. (G) The ROC analysis of RMST. ns, p ≥ 0.05, **p < 0.01, and ***p < 0.001. [file Image_1.TIF]

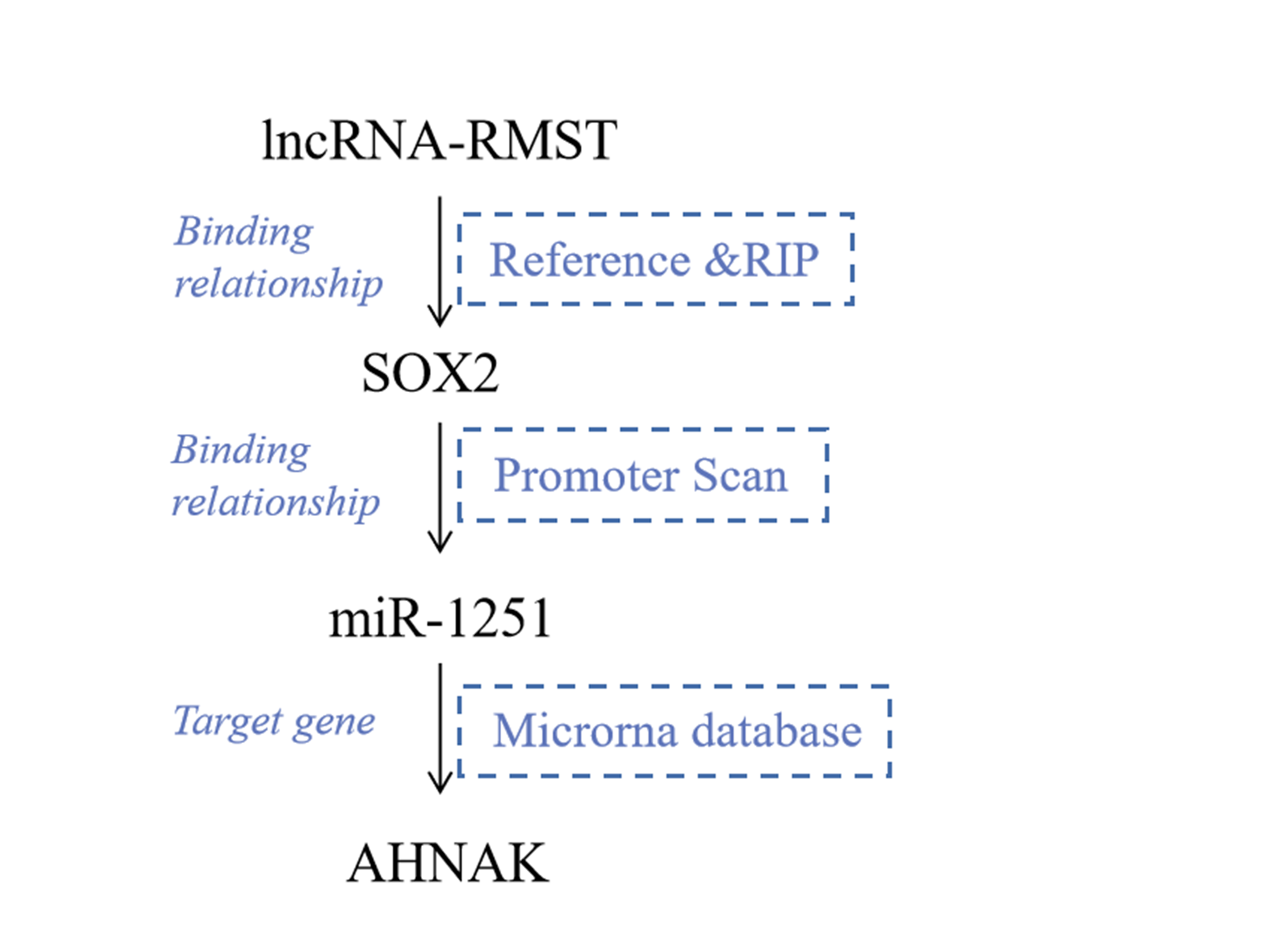

Supplement: Supplementary Figure 2 — The flowchart of bioinformatics analysis steps. The binding relationship between RMST and SOX2 was demonstrated by reference and RIP assay. Promoter Scan was applied to predict the binding relationship between SOX2 and miR-1251 promoter region. MicroRNA database was employed to predict the target gene of miR-1251. [file Image_2.PNG]
